# P-2065. Social Determinants of Health Affecting Management of Care in Patients with Infective Endocarditis

**DOI:** 10.1093/ofid/ofaf695.2229

**Published:** 2026-01-11

**Authors:** Winston D Fu, Allison N Davis, Madeline King, Lisa Pedroza, Henry S Fraimow, Carlo Foppiano Palacios

**Affiliations:** Cooper Medical School of Rowan University; Cooper Medical School of Rowan University; Cooper University Hospital, Camden, New Jersey; Cooper Medical School Rowan University, Camden, New Jersey; Cooper University Hospital and Cooper Medical School of Rowan University, Camden, NJ; Cooper University Health Care, Camden, New Jersey

## Abstract

**Background:**

Patients with infective endocarditis (IE) are at high risk of morbidity, including metastatic spread to other sites. However, certain social determinants of health (SDH) place certain patients at a higher risk of incomplete care and worse long term health outcomes. We sought to evaluate various SDH and their impact on the treatment and outcomes of patients with IE.

**Figure:**
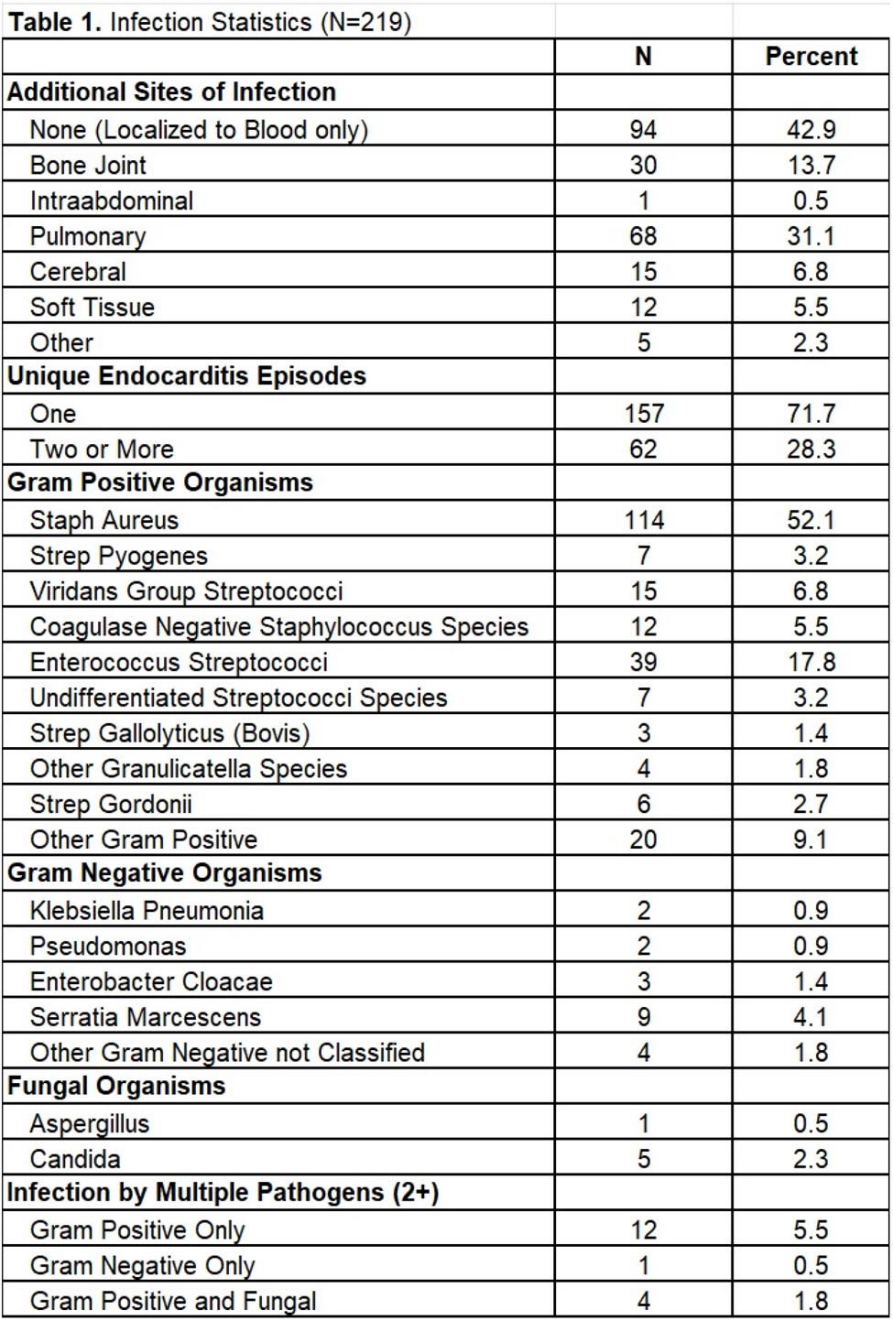

**Methods:**

We conducted a retrospective chart review of adults admitted with blood culture positive IE from 2018-2024 at Cooper University Hospital. A single endocarditis episode included first and subsequent hospitalizations for IE, unless a new infection was suspected. Patients were excluded under 18 years old or if they received treatment for IE, but blood cultures were negative throughout the entire episode. We collected demographics, risk factors for IE, SDH documented on chart, infection details, treatment, and discharge outcomes. Descriptive statistics and Fisher’s exact test were used.

**Results:**

219 patients were included, median age was 42 years, 58% were male, 75% were White, 12% were Black, and 6% were Latino. Most common pathogen was S. Aureus (52%). SDH were prevalent: 64% with history of drug use, 65% on Medicaid, and 40% with housing insecurity. 27% underwent cardiac surgery and 26% left via patient-directed discharge (PDD) at least once during their episode. Furthermore, black patients (p=0.0021), those with housing insecurity (p=< 0.001), and those who left via PDD (p=< 0.001) were less likely to receive cardiac surgery versus all other races/ethnicities. Hospital mortality was more common in Black patients (p=0.04) compared to other races/ethnicities.

**Conclusion:**

We found significant SDH disparities with cardiac and mortality outcomes. Future studies should evaluate implementing certain strategies to address these disparities to improve care and reduce morbidity among vulnerable populations.

**Disclosures:**

Madeline King, PharmD, Innoviva: Advisor/Consultant|Shionogi: Advisor/Consultant

